# ERDRP-0519 inhibits feline coronavirus in vitro

**DOI:** 10.1186/s12917-022-03153-3

**Published:** 2022-01-25

**Authors:** Michele Camero, Gianvito Lanave, Cristiana Catella, Maria Stella Lucente, Alessio Sposato, Viviana Mari, Maria Tempesta, Vito Martella, Alessio Buonavoglia

**Affiliations:** 1 Department of Veterinary Medicine, University of Bari, Valenzano, Italy; 2 Freelance, Bari, Italy

**Keywords:** Feline coronavirus (FCoV), Feline infectious peritonitis (FIP), Cat, Antiviral, ERDRP-0519

## Abstract

**Background:**

Coronaviruses (CoVs) are major human and animal pathogens and antiviral drugs are pursued as a complementary strategy, chiefly if vaccines are not available. Feline infectious peritonitis (FIP) is a fatal systemic disease of felids caused by FIP virus (FIPV), a virulent pathotype of feline enteric coronavirus (FeCoV). Some antiviral drugs active on FIPV have been identified, but they are not available in veterinary medicine. ERDRP-0519 (ERDRP) is a non-nucleoside inhibitor, targeting viral RNA polymerase, effective against morbilliviruses in vitro and in vivo.

**Results:**

The antiviral efficacy of ERDRP against a type II FIPV was evaluated in vitro in Crandell Reese Feline Kidney (CRFK) cells. ERDRP significantly inhibited replication of FIPV in a dose-dependent manner. Viral infectivity was decreased by up to 3.00 logarithms in cell cultures whilst viral load, estimated by quantification of nucleic acids, was reduced by nearly 3.11 logaritms.

**Conclusions:**

These findings confirm that ERDRP is highly effective against a CoV. Experiments will be necessary to assess whether ERDRP is suitable for treatment of FIPV in vivo.

**Supplementary Information:**

The online version contains supplementary material available at 10.1186/s12917-022-03153-3.

## Background

Coronaviruses (CoVs) (family *Coronaviridae*) are enveloped, single-stranded, positive-sense RNA viruses infecting a large variety of animal hosts. CoVs are currently classified within four genera, *Alphacoronavirus, Betacoronavirus, Gammacoronavirus* and *Deltacoronavirus*. CoVs are responsible for diarrhea in cattle and pigs and upper respiratory diseases in chickens [1]. In humans, CoVs cause mainly respiratory tract infections, with mild clinical signs (i.e. the common cold) with exception of hypervirulent CoVs, i.e. Severe Acute Respiratory Syndrome (SARS) CoV-1, Middle East Respiratory Syndrome (MERS) CoV and SARS CoV-2 infectious agent of Coronavirus Disease 2019 (COVID-19), that may cause severe pneumonia requiring hospitalisation and admission to intermediate or intensive care units.

A member of the genus *Alphacoronavirus*, Feline CoV (FCoV), infects cats worldwide. There are two distinct types of FCoV, namely type I FCoV (FCoV-I) and type II FCoVs (FCoV-II), with the latter being derived by recombination between FCoV-I and canine CoV (CCoV) [2]. FCoV exists as two different biotypes, i.e. feline enteric CoV (FeCoV) and feline infectious peritonitis virus (FIPV) [3]. FeCoV causes mild enteritis (usually subclinical infection) whilst FIPV causes a highly lethal systemic disease, due to mutations of the FeCoV pathotype. Several serological and genetic investigations reported that FCoV-I is more prevalent than FCoV-II, and therefore most FIP cases are caused by FCoV-I infection [4, 5]. The disease occurs most commonly in young cats, often less than 1 year of age. FIP is usually diagnosed clinically after the development of effusion in the abdominal and, less frequently, in the pleural cavity and/or the formation of granulomas. Granulomatous lesions are often observed on the surface of numerous organs, including the omentum, intestine, liver, kidney, spleen and lungs [6]. The mortality rate of cats exhibiting these symptoms is high, although some cats can live with the disease for weeks, months or, rarely, years [7].

Since FIP is common in cats, with limited therapeutic and prevention strategies, there is an increasing demand for therapies from veterinary practitioners and cat owners. Likewise, the emergence of hypervirulent human CoVs in the last two decades has prompted the research of antivirals and vaccines against CoVs in human medicine [8, 9].

The most commonly available antiviral drug for the treatment of FIP is feline recombinant interferon omega (Virbagen Omega, Virbac), although the efficacy of interferon has not been demonstrated firmly [10]. Moreover, chloroquine has been shown to inhibit FIPV replication in vitro although it was associated with an untoward toxic effect [11]. Some antivirals, such as the nucleoside analogue GS-441524 (active forms of remdesivir triphosphate, GS) and GC-364 (3C-like protease inhibitor, GC) [12, 13] have been shown to be effective in treating cats with FIP. However, many of them are expensive and/or not available in veterinary medicine [14].

ERDRP-0519 (ERDRP) is a non-nucleoside inhibitor of the RNA-dependent of RNA polymerase (RdRp), an enzyme essential for viral replication. ERDRP has been shown to be effective in both in vitro and in vivo studies. This compound showed promising results in vitro against measles virus (MeV) and canine distemper virus (CDV) and in vivo in CDV-infected ferrets [15, 16]. Although the RdRp gene of CoVs (RNA+) and paramyxoviruses (RNA-) shows extensive sequence divergence with different evolutionary patterns [17], the RdRps share several conserved motifs required for polymerase functionality [18]. Accordingly, it is possible that molecule able to interfere with the RdRp activity of RNA- viruses could also affect the RdRp of RNA+ viruses, interacting with highly conserved residues in linear or structural active sites. Other non-nucleoside RDRP inhibitors tested in vitro against coronaviruses have shown promising results [19, 20]. Therefore we hypothesized that ERDRP could exert antiviral activity against CoVs and we evaluated in vitro the antiviral effects using FIPV as virus model.

## Results

### Cytotoxicity assay

Cytotoxicity was evaluated by the In vitro Toxicology Assay Kit (Sigma–Aldrich Srl, Milan, Italy), based on 3-(4,5-dimethylthiazol-2 yl) -2,5-diphenyl tetrazolium bromide (XTT) after exposing Crandell Reese Feline Kidney (CRFK) cells to ERDRP at various concentrations (10, 20, 30, 40, 45, 60 and 70 μM) for 72 h. The intensity and variety of the cellular morphological changes (loss of cell monolayer, granulation, vacuolization in the cytoplasm, stretching and narrowing of cell extensions and darkening of the cell borders) were dose-dependent and cytotoxicity was assessed by measuring spectrophotometrically the absorbance signal. Based on fitted dose–response curves, the CC_20_ of ERDRP was set at 50 μM. In all the experiments, dimethyl sulfoxide (DMSO), used as vehicle control, did not show any cpe on cells.

**Fig. 1:**
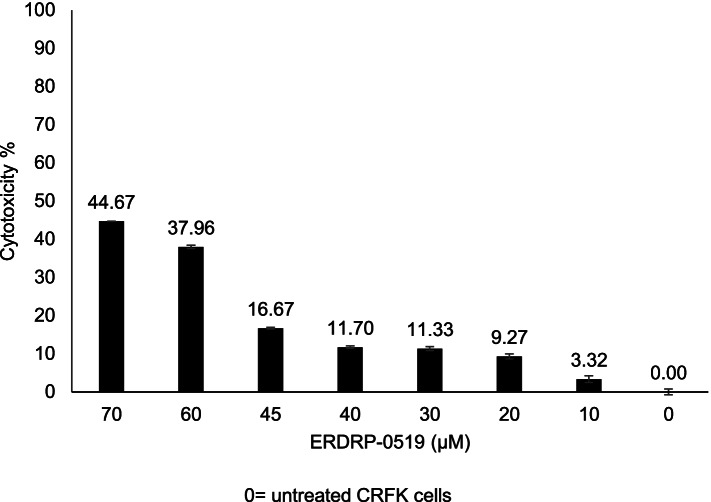
Cytotoxicity of the CRFK cells treated with ERDRP-0519 (ERDRP) and calculated after 72 h post treatment by XTT assay. The value was calculated setting as 0% the cytotoxicity untreated cells. Cytotoxicity is plotted against different concentrations (μM) of ERDRP. Bars in figures indicate the means. Error bars indicate the standard deviation

When comparing the cytotoxicity on the treated cells of the compound at concentrations below CC_20_ (45, 40, 30, 20 and 10 μM), the one-way Analysis of Variance (ANOVA) model showed a statistically significant decrease in cytotoxicity (F = 201.2, *p* < 0.0001). By a two-by-two comparison of individual ERDRP concentrations (45, 40, 30, 20 and 10 μM) statistically significant decreases in cytotoxicity were observed (*p* < 0.0001) and only the comparison between the concentrations 40 μM and 30 μM was not statistically significant (*p* = 0.9340) (Supplementary Table 1). Consequently, the experiments to assess the antiviral activity were carried out using concentrations of drugs below the cytotoxic threshold, starting from 50 μM. Untreated cells were used in each experiment as negative control and considered as 0% cytotoxicity. Cytotoxicity, expressed as a percentage, was calculated based on cytotoxicity of ERDRP on the CRFK cells and plotted against the drug concentrations (Fig. 1). Cytotoxicity of the CRFK cells treated with ERDRP at the higher concentrations (70, 60 μM) ranged from 44.67 to 37.96%, and decreased from 16.67 to 3.32% at the lower concentrations (45, 40, 30, 20 and 10 μM) (Supplementary Table 1).

### Antiviral activity assay

For the replication inhibition assays, CRFK cells were infected with 20 Tissue Culture Infectious Dose (TCID_50_) of FCoV-II strain 25/92. Antiviral activity of ERDRP against the virus was tested at different concentrations chosen based on the cytotoxicity assay results, starting from 50 μM (CC_20_) down to 45, 40, 30, 20 and 10 μM. Viral titres, were evaluated by endpoint dilution method (observation of cpe in cell monolayers) and viral nucleic acid (NA) copies/10 μl were calculated by NA quantification using reverse-transcriptase (RT) quantitative PCR (RT-qPCR).

**Fig. 2:**
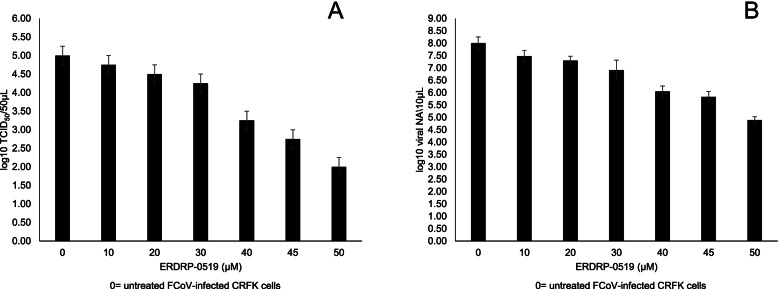
Viral titres of the supernatants collected at 72 h post infection of FCoV-infected CRFK cells untreated and treated with ERDRP-0519 (ERDRP). The viral titers, expressed as log10 TCID_50_/50 μl, were plotted against various non-cytotoxic concentrations (10 to 50 μM) of ERDRP. **A**. Viral nucleic acids (NA) copies measured in 10 μl of the supernatants collected at 72 h post infection from CRFK cells infected with FCoV, either untreated or treated with ERDRP. The viral NA copies, expressed as log10 viral NA/10 μl were plotted against various non-cytotoxic concentrations (10 to 50 μM) of ERDRP (**B**). Bars in the figures indicate the means. Error bars indicate the standard deviation

Viral titres of the cells treated with the compound were expressed as log10 TCID_50_/50 μl and plotted against the non-cytotoxic drug concentrations (Fig. 2A). Comparisons between untreated (mean = 5 log10 TCID_50_/50 μl, standard deviation (SD) = 0.25 log10 TCID_50_/50 μl) and ERDRP treated infected cells revealed a statistically significant average decrease of 0.75 log10 TCID_50_/50 μl at 30 μM (*p* = 0.0314, 95% confidence interval (95% CI) = [0.053; 1.447]), 1.75 log10 TCID_50_/50 μl at 40 μM (*p* < 0.0001, 95% CI = [1.053; 2.447]), 2.25 log10 TCID_50_/50 μl at 45 μM (*p* < 0.0001, 95% CI = [1.553; 2.947]) and of 3.00 log10 TCID_50_/50 μl at 50 μM (*p* < 0.0001, 95% CI = [2.303; 3.697]). ERDRP at 10 and 20 μM also determined an average decrease in the viral titre of 0.25 (*p* = 0.8733, 95%CI = [− 0.447; 0.947]) and 0.50 (*p* = 0.2486, 95%CI = [− 0,197; 1197]) log10 TCID_50_/50 μl compared to untreated infected cells although without any statistical significance (Fig. 2A) (Supplementary Table 2).

Viral NAs were expressed as log10 viral NA copies/10 μl of infected cells treated with the compound and of untreated infected cells and plotted against the non-cytotoxic drug concentrations (Fig. 2B).

The comparison between untreated infected cells (mean = 8.00 log10 viral NA copies/10 μl, SD = 0.25 log10 viral NA copies/10 μl) with cells treated with ERDRP at 20 μM (mean = 7.29 log10 viral NA copies/10 μl, SD = 0.18 log10 viral NA copies/10 μl), at 30 μM (mean = 6.91 log10 viral NA copies/10 μl, SD = 0.40 log10 viral NA copies/10 μl), at 40 μM (mean = 6.05 log10 viral NA copies/10 μl, SD = 0.22 log10 viral NA copies/10 μl), at 45 μM (mean = 5.83 log10 viral NA copies/10 μl, SD = 0.22 log10 viral NA copies/10 μl) and at 50 μM (mean = 4.89 log10 viral NA copies/10 μl, SD = 0.14 log10 viral NA copies/10 μl), revealed statistically significant average decreases in viral load of 0.7133 (*p* = 0.0087, 95% CI = [0.1568; 1.270], 1.097 (*p* = 0.0002, 95% CI = [0.5401; 1.653], 1.96 (*p* < 0.0001, 95% CI = [1.403; 2517], 2.177 (*p* < 0.0001, 95% CI = [1.620; 2.733] and 3.113 (*p* < 0.0001, 95% CI = [2.557; 3.670] log10 viral NA copies/10 μl, respectively. ERDRP at 10 μM (mean = 7.48 log10 viral NA copies/10 μl, SD = 0.23 log10 viral NA copies/10 μl,) determined a slight decrease of 0.5267 log10 viral NA copies/10 μl (*p* = 0.0691, 95% CI = [− 0.02987; 1.083) compared to untreated infected cells, although without any statistical significance (Fig. 2B) (Supplementary Table 1).

The ANOVA model showed a statistically significant effect of treatment in the comparison based on the viral titration (F = 61.43, *p* < 0.0001) and in the comparison based on viral NA quantification (F = 89.49, *p* < 0.0001). Virus growth in CRFK cells was affected to various extents by the concentrations of the molecule tested in this study.

Based on viral titration, the Selectivity index (SI) of ERDRP on CRFK cells after 72 h of exposure was assessed at 0.75 and calculated as CC_20_/IC_80_ (50.00/66.08 μM). Based on viral DNA quantification, the SI of ERDRP on CRFK cells after 72 h of exposure was assessed at 0.64 and calculated as CC_20_/IC_80_ (50.00/77.41 μM).

## Discussion

RNA viruses greately differ in terms of virion structure and genome organization, mechanisms of entry and assembly during cell replication. However, fundamental features in their genome replication and transcription are shared across different RNA viruses, with the virally encoded RdRp processing the biosynthesis of an RNA product directed by an RNA template. Viral RdRps greatly vary in size and structural organization [21–25]. However, all RdRPs share a 50- to 70-kDa polymerase core that forms a unique encircled right-hand structure with palm, fingers, and thumb domains. The RdRP catalytic motifs are located within the most conserved palm domain and in the fingers, arranged around the active site [26–29], including the RdRp of CoVs [30] and the RdRp domain of mononegaviruses [31]. The structural conservation of the RdRP polymerase core and the active motifs form the basis for understanding the common features in viral RdRP catalytic mechanisms and for developing strategies targeting the RdRp with possible broad-spectrum potential.

Based on this assumption, we tested ERDRP, a non-nucleoside inhibitor of viral RdRp, against FIPV in vitro. ERDRP targets morbillivirus L protein, the catalytically active subunit of the polymerase complex, inhibing all phosphodiester bond formation in both de novo initiation of RNA synthesis at the promoter and RNA elongation by a committed polymerase complex [15, 32]. ERDRP has shown inhibitory activity on measles virus and canine distemper virus (CDV) but not against respiratory scincizial virus, a non-morbillivirus paramyxovirus [15], and therefore it was believed to have morbillivirus-specific spectrum. In our study, however, ERDRP was able to inhibit replication of the feline CoV FIPV in a dose-dependent fashion, reducing viral titer by up to 3 log10 and viral load by up to 3.11 log10 viral NA copies/10 μl at 50 μM (Fig. 2). These findings extend the spectrum of activity of this class of RdRp inhibitors to a phylogenetically unrelated RNA virus. Likewise, it could be interesting to test also the activity for CoVs of other classes of non-nucleside inhibitors.

In previous studies ERDRP, used at higher concentrations (up to 100 μM), showed a low cytotoxicity in Vero (African green monkey kidney epithelial) [15, 32] and baby hamster kidney (BHK-21) cells and human peripheral blood mononuclear (PBMCs), embryonic kidney 293 and epithelioma-2 cells [15]. In our study, we assessed the maximum non cytotoxic (CC_20_) concentration of ERDRP at 50 μM and we observed significant antiviral activity until 30 μM. Antivirals tested effective at lower dosages would allow reducing possible toxic effects in prolonged therapies. Krumm et al. [15] reported excellent biodisponibility of ERDRP after oral administration in CDV-infected ferrets, thus representing a proof of concept of its possible therapeutic development [15]. Prophylactic oral treatment with ERDRP of ferrets infected intranasally with a lethal CDV dose reduced viremia and prolonged survival of animals. CDV-infected ferrets receiving post-infection treatment with ERDRP at the onset of viremia showed low viral loads, remained asymptomatic and recovered from infection. Recovered animals also mounted a robust immune response and were protected against re-challenge with a lethal CDV dose [15].

Analogous proof-of-concepts animal experiments could be proposed to assess the therapeutic potential of ERDRP in treatment of FIP in cats.

FIP is a frustrating disease for practitioners and represents a painful and unacceptable diagnosis for cat owners. Since there are no relevant antigenic differences between enteric FeCoV and hypervirulent FIP strains, the detection of elevated antibody titers alone is not a confirmatory test [33]. Cats suffering from FIP are known to develope a significant humoral response to the virus rather than a cell-mediated immune response which may contribute to the pathogenesis of FIP [34]. FIP is an example of a viral disease, in which serum antibodies, rather than being protective, increases the severity of the infection with antibody-dependent enhancement (ADE) mechanisms [35, 36]. Prophylaxis based on vaccines proved ineffective in protecting cats and was associated to ADE-mediated adverse effects [35]. A modified live intranasal vaccine authorized in the United States for the prevention of FIP is not recommended by the American Association of Feline Practitioners [37].

Overall, FIP is perceived as a real threat to feline health and, to date, therapy is mainly based on the control of clinical signs. Supportive corticosteroid treatment in cats is administered to suppress the inflammatory immune response [38]. Unfortunately, however, specific antiviral molecules able to limit FeCV replication in infected cats, are not available, despite the relentless demand from veterinarians and pet owners for life-saving specific antiviral therapies. Some antivirals such as GS and GC [12, 13] proved to be effective in treating cats with FIP but they have not been licensed for use in veterinary medicine [14]. GS causes a rapid reversal of disease signs and recovery in cats infected experimentally with FIPV [39] and it has been evaluated experimentally in field trials [13]. The lack of effective therapies for cats with FIP has fuelled the black market (http://www.catvirus.com/downloads/Dr. Pedersen Statement on GS and GC.pdf). Importantly, GS has been used in COVID-19 patients against SARS-CoV-2 [40] whilst its development for therapy in cats with FIP has been abandoned. Interstingly, this parallelism between FIP and COVID-19 sets cats as a possible model for the study of antivirals against CoVs.

CoVs are posing a number of challenges for human and veterinary medicine, and different strategies, eventually combined, are required to counteract adeguately and minimize their impact on human and animal health. Exploring the antiviral effects of the vast repertoire of drugs already developed and licensed or under development could be helpful to obtain novel effective tools against CoVs.

## Methods

### Cells and virus

CRFK cells [American Type Culture Collection (ATCC) CCL-94TM, Manassas, Virginia, USA)] were cultured at 37 °C in a 5% CO_2_ atmosphere in Dulbecco-MEM (D-MEM) supplemented with 10% foetal bovine serum, 100 IU / ml penicillin, 0.1 mg/ml streptomycin and 2 mM l-glutamine. The same medium was used for the antiviral assays. FCoV-II strain 25/92 isolated from a dead cat with infectious peritonitis was cultured and titrated in CRFK cells [41]. The virus stock with a titre of 10^5.75^ TCID_50_/50 μl was stored at − 80 °C and used for the experiments.

### Antiviral molecules

ERDRP (1-Methyl-N-[4-[[(2S) -2-[2-(4-morpholinyl) ethyl] -1-piperidinyl] sulfonyl]phenyl]-3-(trifluoromethyl)-1H-pyrazole-5-carboxamide) (Aobious Inc., Gloucester, Massachusetts, USA) was tested against the virus. ERDRP was initially diluted in DMSO (Sigma-Aldrich, St. Louis, Missouri, USA) to obtain a stock concentration of 9.44 mM and stored at − 80 °C until use.

### Cytotoxicity assay

Cytotoxicity of ERDRP was assessed by XTT assay [42] using the In Vitro Toxicology Assay Kit (Sigma–Aldrich Srl, Milan, Italy), based on 3-(4,5-dimethylthiazol-2yl)-2,5-diphenyl tetrazolium bromide (XTT). Confluent 24-h monolayers of CRFK cells grown in 96-well plates were used to assess the cytotoxicity of ERDRP at different concentrations (20, 30, 40, 45, 60 and 70 μM).

In all experiments, untreated cells and cells treated with equivalent dilutions of DMSO without ERDRP were used as negative control and vehicle control, respectively. After 72 h of incubation, XTT stock solution (70 μl, 70% of the total cell volume) was added to each well and the plates were incubated at 37 °C. After 4 h the plates were read in an automatic spectrophotometer (microtitre plate absorbance reader iMark Bio-Rad) at a test wavelength of 450 nm (A_450_) and a background wavelength of 655 nm (A_655_). The final absorbance was calculated as A_450_- A_655_.

The absorbance of negative control was set as 0% cytotoxicity and the values for treated cells were calculated as follows: % cytotoxicity = [((A_450_ - A_655_) negative control-(A_450_ - A_655_) treated cells) / (A_450_ - A_655_) negative control] × 100% [43]. The experiments were performed in triplicate.

Moreover to evaluat cell viability the monolayers treated with ERDRP at maximum concentration have been subjected to they were trypsinized for further cellular passages.

### Antiviral activity assay

Based on the cytotoxicity assay results, the antiviral activity against the FCoV-II strain 25/92 was evaluated using ERDRP at CC_20_ = 50 μM and below the cytotoxic threshold.

The antiviral activity of ERDRP against the virus was evaluated at different concentrations (10, 20, 30, 40, 45 and 50 μM) in three independent experiments. Confluent monolayers of CRFK cells of 24 h in 24-well plates were infected with 100 μl of the virus containing 20 TCID_50_, with a Multiplicity of Infection (MOI) of 0.45. After virus adsorption for 1 h at 37 °C, the inoculum was removed, the monolayers were washed once with D-MEM and 1 ml of ERDRP was added. In the untreated infected cells, D-MEM was used to replace the inoculum [43].

After 72 h, aliquots of supernatants from ERDRP-treated and -untreated infected cells were collected for subsequent viral titration and for nucleic acids (NAs) detection and quantification.

### Viral titration

Ten-fold dilutions of the supernatants of untreated infected cells and of cells treated with ERDRP were titrated in quadruplicates in 96-well plates containing CRFK cells. The plates were incubated for 72 h at 37 °C in 5% CO_2_ and the viral titres were determined based on the cytopathic effect (cpe) observation [43].

### Detection of FCoV NAs

For FCoV NAs detection, 140 μl of the supernatants were used for RNA extraction by means of QIAamp® Viral RNA Mini Kit (Qiagen S.p.A., Milan, Italy), following the manufacturer’s protocol and the NA templates were stored at − 70 °C until their use. FCoV RT-qPCR was performed as previously described [44], with minor modifications. In brief, a one-step method was adopted using Platinum® Quantitative PCR SuperMix-UDG (Invitrogen srl, Milan, Italy) and the following 50-μl mixture: 25 μl of master mix, 300 nM of primers FcoV1128f (GATTTGATTTGGCAATGCTAGATTT) and FcoV1229r (AACAATCACTAGATCCAGACGTTAGCT), 200 nM of probe FCoV1200p (FAM- TCCATTGTTGGCTCGTCATAGCGGA-BHQ1) and 10 μl of template NA. The employed oligonucleotides bind to the 3′ untranslated region (UTR) [44]. The thermal profile consisted of incubation with Uracil DNA glycosylase (UDG) at 50 °C for 2 min and activation of Platinum Taq DNA polymerase at 95 °C for 2 min, followed by 45 cycles of denaturation at 95 °C for 15 s, annealing at 48 °C for 30 s and extension at 60 °C for 30s. Tenfold serial dilutions of the FCoV standard 3′ UTR NA, representing 10^0^ to 10^8^ copies of viral NA/10 μl of template, were made out in Tris–HCl, EDTA (TE) buffer. Aliquots of each dilution were frozen at − 80 °C and used only once.

### Data analysis

After logarithmic conversion of ERDRP concentrations, the data obtained in the cytotoxicity and antiviral activity assays were analysed by a non-linear curve fitting procedure. The goodness of fit was tested by non-linear regression analysis of the dose-response curve. From the fitted dose–response curves obtained in each experiment, the non-cytotoxic concentration (CC_20_) was defined as the concentration at which viability of the treated cells decreased to no more than 20% compared to the control cells. The antiviral activity was expressed as the concentration required to reduce virus replication by 80% (IC_80_) in the treated cells compared with the untreated infected cells. The CC_20_ and IC_80_ values were calculated as the mean ± SD of three experiments. SI was calculated by CC_20_ in CRFK cells/IC_80_ against FCoV [43].

Data from cytotoxicity and antiviral activity assays were expressed as mean ± standard deviation (SD). Shapiro-Wilk test was used to assess the normality of distribution. Data were analysed for the effect of drug concentration by ANOVA using Tukey’s test as post hoc test (statistical significance set at 0.05) and 95% CI were calculated [43].

Statistical analyses were performed with the software GraphPad Prism v 8.0.0 (GraphPad Software, San Diego, CA, USA).

## Supplementary Information

**Additional file 1.**

**Additional file 2.**

## Data Availability

All data generated or analysed during this study are included in this published article [and its supplementary information files].

## Abbreviations

CoV: Coronavirus
FIP: feline infectious peritonitis
FIPV: feline infectious peritonitis virus
FeCoV: feline enteric coronavirus
ERDRP: ERDRP-0519
CRFK: Crandell Reese Feline Kidney
SARS: Severe Acute Respriratory Syndrome
MERS: Middle East Respiratory Syndrome
FCoV: Feline coronavirus
CCoV: Canine coronavirus
COVID-19: Coronavirus Disease 2019
GS: GS-441524
GC: GC-364
RdRp: RNA-dependent RNA polymerase
MeV: Measles virus
CDV: Canine distemper virus
XTT: In vitro Toxicology Assay Kit based on 3-(4,5-dimethylthiazol-2 yl) -2,5-diphenyl tetrazolium bromide
DMSO: Dimethyl sulfoxide
TCID_50_: Tissue Culture Infection Dose
RT: Reverse-transcriptase
RT-qPCR: Reverse-transcriptase quantitative PCR
SI: Selectivity index
SD: Standard deviation
95% CI: 95% confidence interval
WHO: World Health Organisation
CMI: Cell-mediated immune
D-MEM: Dulbecco Minimal Essential Medium
MOI: Multiplicity of Infection
cpe: Cytopathic effect
UTR: Untranslated region
UDG: Uracil DNA glycosylase
TE: Tris–HCl, EDTA
