# Supplementary material for: ERDRP-0519 inhibits feline coronavirus in vitro
Source: BMC Vet Res. 2022 Jan 25;18:55. doi: 10.1186/s12917-022-03153-3 (PMC8787031; doi:10.1186/s12917-022-03153-3)
Supplement: Supplementary file 1 — Additional file 1. [file 12917_2022_3153_MOESM1_ESM.docx]

**Table 1.** Comparison of cytotoxicity activity of ERDRP-0519 in CRFK cells treated with the compound at different concentrations (10, 20, 30, 40, 45, 60 and 70μM) and with untreated cells (UC).

| Comparisons | Cytotoxicity % | | |
| --- | --- | --- | --- |
|  | MDC | 95% CI | P Value |
| UC vs 10µM | -2.97 | [-3.373; -2.567] | <0.0001*** |
| 10µM vs 20µM | -5,95 | [-6.353; -5.547] | <0.0001*** |
| 10µM vs 30µM | -8.01 | [-8.413; -7.607] | <0.0001*** |
| 10µM vs 40µM | -8.38 | [-8.783; -7.977] | <0.0001*** |
| 10µM vs 45µM | -13.35 | [-13.75; -12.95] | <0.0001*** |
| 10µM vs 60µM | -34.64 | [-35.04; -34.24] | <0.0001*** |
| 10µM vs 70µM | -41.35 | [-41.75; -40.95] | <0.0001*** |
| UC vs 20µM | -8.92 | [-9.323; -8.517] | <0.0001*** |
| 20µM vs 30µM | -2.06 | [-2.463; -1.657] | <0.0001*** |
| 20µM vs 40µM | -2.43 | [-2.833; -2.027] | <0.0001*** |
| 20µM vs 45µM | -7.40 | [-7.803; -6.997] | <0.0001*** |
| 20µM vs 60µM | -28.69 | [-29.09; -28-29] | <0.0001*** |
| 20µM vs 70µM | -35.40 | [-35.80; -35.00] | <0.0001*** |
| UC vs 30µM | -10.98 | [-11.38; -10.58] | <0.0001*** |
| 30µM vs 40µM | -0.37 | [-0.773; 0.0325] | 0.0837 ns |
| 30µM vs 45µM | -5.34 | [-5.743; -4.937] | <0.0001*** |
| 30µM vs 60µM | -26.63 | [-27.03; -26.23] | <0.0001*** |
| 30µM vs 70µM | -33.34 | [-33.74; -32.94] | <0.0001*** |
| UC vs 40µM | -11.35 | [-11.75; -10.95] | <0.0001*** |
| 40µM vs 45µM | -4.97 | [-5.373; -4.567] | <0.0001*** |
| 40µM vs 60µM | -26.26 | [-26.66; -25.86] | <0.0001*** |
| 40µM vs 70µM | -32.97 | [-33.37; -32.57] | <0.0001*** |
| UC vs 45µM | -16.32 | [-16.72; -15.92] | <0.0001*** |
| 45µM vs 60µM | -21.29 | [-21.69; -20.89] | <0.0001*** |
| 45µM vs 70µM | -28.00 | [-28.40; -27.60] | <0.0001*** |
| UC vs 60µM | -37.61 | [-38.01; -37.21] | <0.0001*** |
| 60µM vs 70µM | -6.71 | [-7.113; -6.307] | <0.0001*** |
| UC vs 70µM | -44.32 | [-44.72; -43.92] | <0.0001*** |

**Legend**: MDC: mean difference of cytotoxicity; 95% CI: 95% confidence interval; ns: not significant; * significant; **very significant; ***highly significant
