# Supplementary material for: ERDRP-0519 inhibits feline coronavirus in vitro
Source: BMC Vet Res. 2022 Jan 25;18:55. doi: 10.1186/s12917-022-03153-3 (PMC8787031; doi:10.1186/s12917-022-03153-3)
Supplement: Supplementary file 2 — Additional file 2. [file 12917_2022_3153_MOESM2_ESM.docx]

**Table 2.** Comparison of antiviral activity of ERDRP-0519 against FCoV-II strain 25/92 in infected cells treated with the compound at different concentrations (10, 20, 30, 40, 45 and 50µM) and with untreated infected cells (CV).

| Comparisons | Viral titers (log10 TCID50/50µl) | | | Viral nucleic acids (log10 viral DNA copy number/10µl) | | |
| --- | --- | --- | --- | --- | --- | --- |
|  | MDV | 95% CI | P Value | MDN | 95% CI | P Value |
| CV vs 10µM | 0.25 | [-0.447; 0.947] | 0.8733 ns | 0.5267 | [-0.02987; 1.083] | 0.0691 ns |
| 10µM vs 20µM | 0.25 | [-0.447; 0.947] | 0.8733 ns | 0.1867 | [-0.3699; 0.7432] | 0.903 ns |
| 10µM vs 30µM | 0.50 | [-0.197; 1.197] | 0.2486 ns | 0.5700 | [0.01346; 1.127] | 0.0431* |
| 10µM vs 40µM | 1.50 | [0.803; 2.197] | <0.0001*** | 1.433 | [0.8768; 1.990] | <0.0001*** |
| 10µM vs 45µM | 2.00 | [1.303; 2.697] | <0.0001*** | 1.650 | [1.093; 2.207] | <0.0001*** |
| 10µM vs 50µM | 2.75 | [2.053; 3.447] | <0.0001*** | 2.587 | [2.030; 3.143] | <0.0001*** |
| CV vs 20µM | 0.50 | [-0.197; 1.197] | 0.2486 ns | 0.7133 | [0.1568; 1.270] | 0.0087** |
| 20µM vs 30µM | 0.25 | [-0.447; 0.947] | 0.8733 ns | 0.3833 | [-0.1732; 0.9399] | 0.2865 ns |
| 20µM vs 40µM | 1.25 | [0.553; 1.947] | 0.0004*** | 1.247 | [0.6901; 1.803] | <0.0001*** |
| 20µM vs 45µM | 1.75 | [1.053; 2.447] | <0.0001*** | 1.463 | [0.9068; 2.020] | <0.0001*** |
| 20µM vs 50µM | 2.50 | [1.803; 3.197] | <0.0001*** | 2.400 | [1.843; 2.957] | <0.0001*** |
| CV vs 30µM | 0.75 | [0.053; 1.447] | 0.0314* | 1.097 | [0.5401; 1.653] | 0.0002*** |
| 30µM vs 40µM | 1.00 | [0.303; 1.697] | 0.0034** | 0.8633 | [0.3068; 1.420] | 0.0017** |
| 30µM vs 45µM | 1.50 | [0.803; 2.197] | <0.0001*** | 1.080 | [0.5235; 1.637] | 0.0002*** |
| 30µM vs 50µM | 2.25 | [1.553; 2.947] | <0.0001*** | 2.017 | [1.460; 2.573] | <0.0001*** |
| CV vs 40µM | 1.75 | [1.053; 2.447] | <0.0001*** | 1.960 | [1.403; 2.517] | <0.0001*** |
| 40µM vs 45µM | 0.50 | [-0.197; 1.197] | 0.2486 ns | 0.2167 | [-0.3399; 0.7732] | 0.8277 ns |
| 40µM vs 50µM | 1.25 | [0.553; 1.947] | 0.0004*** | 1.153 | [0.5968; 1.710] | <0.0001*** |
| CV vs 45µM | 2.25 | [1.553; 2.947] | <0.0001*** | 2.177 | [1.620; 2.733] | <0.0001*** |
| 45µM vs 50µM | 0.75 | [0.053; 1.447] | 0.0314* | 0.9367 | [0.3801; 1.493] | 0.0008*** |
| CV vs 50µM | 3.00 | [2.303; 3.697] | <0.0001*** | 3.113 | [2.557; 3.670] | <0.0001*** |

**Legend**: MDV: mean difference of viral titers; MDN: mean difference of viral nucleic acids; 95% CI: 95% confidence interval; ns: not significant; * significant; **very significant; ***highly significant
